# Metformin-loaded mesoporous calcium silicate nanoparticles against alkaline-resistant *Enterococcus faecalis* infection

**DOI:** 10.1080/20002297.2026.2753969

**Published:** 2026-11-07

**Authors:** Runze Liu, Mengting Duan, Lian Bai, Zhongyuan Zhan, Yandi Dong, Yi Luo, Wei Fan

**Affiliations:** a The State Key Laboratory of Oral & Maxillofacial Reconstruction and Regeneration, Key Laboratory of Oral Biomedicine Ministry of Education, Hubei Key Laboratory of Stomatology, School & Hospital of Stomatology, Wuhan University, Wuhan, China

**Keywords:** *Enterococcus faecalis*, intracanal medicament, mesoporous calcium silicate nanoparticles, metformin, persistent apical periodontitis

## Abstract

**Background:**

*Enterococcus faecalis* (*E. faecalis*) contributes to persistent endodontic infections through biofilm formation, dentinal tubule invasion, and alkaline resistance, which limits the efficacy of calcium hydroxide. Metformin (Met) attenuates this resistance by interfering with bacterial metabolic adaptation. Combining Met with mesoporous calcium silicate nanoparticles (MCSNs), which possess intrinsic alkaline antibacterial activity and sustained drug-delivery capabilities, may enhance the elimination of alkaline-resistant *E. faecalis* and promote periapical healing.

**Methods:**

Metformin-loaded MCSNs (MCSNs-Met) were characterized for physicochemical properties, Met release, alkalinity, cytocompatibility, and dentinal tubule penetration. Antibiofilm activity against highly alkaline-resistant *E. faecalis* was assessed using infected dentin models. Therapeutic efficacy was further evaluated in a beagle model of *E. faecalis*-induced apical periodontitis using Micro-CT and histological analyses.

**Results:**

MCSNs-Met exhibited a uniform mesoporous structure, sustained Met release and alkalinity, favorable cytocompatibility, and enhanced dentinal tubule penetration. Compared with MCSNs alone, MCSNs-Met more effectively disrupted *E. faecalis* biofilms and reduced viable bacterial signals. *In vivo*, MCSNs-Met produced the greatest reduction in periapical bone resorption, markedly decreased residual bacterial signals and inflammatory infiltration, reduced osteoclast activity, and enhanced collagen deposition and periapical tissue repair.

**Conclusions:**

MCSNs-Met integrates sustained Met delivery with the intrinsic alkaline and bioactive properties of calcium silicate nanoparticles, thereby enhancing control of highly alkaline-resistant *E. faecalis* and promoting periapical healing.

## Introduction

Apical periodontitis is one of the most prevalent inflammatory diseases caused by microbial infection within the root canal system [1,2]. Despite significant advances in contemporary endodontic therapy, persistent microorganisms harbored in the anatomical complexities of the root canal system remain a major contributor to treatment failure and disease recurrence [3]. The complex three-dimensional architecture of root canals, including lateral canals, isthmuses, and dentinal tubules, presents substantial challenges for complete microbial eradication through conventional mechanical instrumentation and chemical irrigation [4]. Notably, microorganisms that infiltrate deep into dentinal tubules can escape antimicrobial interventions and act as persistent reservoirs, thereby sustaining intraradicular infection and impairing periapical healing [3].

Apical periodontitis is widely recognized as a polymicrobial disease characterized by a complex and dynamic microbial community within the root canal system [5]. Following root canal treatment, residual microorganisms may persist despite disinfection, whereas newly introduced microorganisms may establish secondary infection, particularly through coronal leakage. Both can sustain intraradicular infection and trigger periapical inflammation and bone resorption, ultimately contributing to post-treatment apical periodontitis [3]. In these infections, facultative anaerobic and Gram-positive bacteria are frequently identified, with *Enterococcus* species being strongly associated with secondary root canal infection and secondary apical periodontitis [6]. Among these microorganisms, *Enterococcus faecalis* (*E. faecalis*) is considered a key pathogen implicated in persistent endodontic infections owing to its remarkable environmental adaptability, ability to penetrate dentinal tubules, biofilm-forming capacity, and resistance to unfavorable conditions within the root canal environment [7]. Despite thorough chemomechanical preparation, *E. faecalis* may survive within complex root canal anatomies through biofilm formation, which enhances bacterial persistence and resistance to alkaline stress, ultimately contributing to sustained intraradicular infection and endodontic treatment failure [8,9]. Therefore, developing effective intracanal medicaments capable of penetrating deep dentinal tubules and eliminating alkaline-resistant *E. faecalis* remains a critical challenge in endodontic therapy.

Calcium hydroxide (CH) remains the most widely used intracanal medicament due to its potent antibacterial activity, primarily attributed to hydroxyl ion release and the establishment of a highly alkaline microenvironment [10]. However, the therapeutic efficacy of CH against persistent *E. faecalis* infections remains suboptimal [11]. Alkaline-resistant *E. faecalis* refers to strains capable of surviving under elevated-pH conditions. This resistance involves adaptive mechanisms, including the maintenance of intracellular pH homeostasis through membrane-associated proton pumps and metabolic adaptation mediated by Man-PTS EII [12]. Moreover, the buffering effect of dentin can reduce the local antibacterial activity of CH [11], while the relatively large particle size and limited diffusion capacity of CH hinder its penetration into deep dentinal tubules, where residual microorganisms may persist beyond the reach of conventional intracanal medication [13]. Therefore, there is an urgent need to develop advanced intracanal therapeutic strategies that not only enhance antibacterial efficacy but also facilitate deep penetration and sustained antimicrobial activity within the complex root canal system.

Metformin (Met), an FDA-approved metabolic drug, possesses a well-established safety profile, excellent cost-effectiveness, and emerging therapeutic potential in the management of oral diseases [14–16]. Our previous study demonstrated that Met could attenuate the alkaline resistance of *E. faecalis* by disrupting bacterial metabolic adaptation mediated by the mannose phosphotransferase system (Man-PTS EⅡ), thereby enhancing the antibacterial efficacy of CH [12]. However, despite its promising antimicrobial activity, the clinical translation of Met-based intracanal therapy remains challenging due to its insufficient retention and rapid diffusion within the root canal environment [13]. Furthermore, whether Met-containing formulations can effectively penetrate dentinal tubules and maintain therapeutically relevant concentrations within these anatomical niches remains largely unknown.

Mesoporous calcium silicate nanoparticles (MCSNs) have emerged as promising multifunctional biomedical materials owing to their high specific surface area, well-defined mesoporous structure, excellent drug-loading capacity, and sustained release properties [17,18]. Importantly, MCSNs are not merely passive drug carriers. Their intrinsic alkalinity can exert antibacterial effects within the root canal environment, while their mesoporous architecture facilitates penetration into dentinal tubules and sustained local drug delivery [19,20]. Moreover, ultrasonic activation can enhance the dispersion and penetration of MCSNs into complex root canal anatomies, including irregularities and dentinal tubules, thereby facilitating their localized and sustained antimicrobial effects [20]. In addition, the release of calcium and silicate ions may promote mineralized matrix deposition and periapical tissue repair [19,20]. Therefore, MCSNs may provide both intrinsic antibacterial activity and an effective platform for enhancing the penetration, retention, and local efficacy of Met. Nevertheless, their inherent antibacterial activity remains insufficient against highly alkaline-resistant microorganisms, partly due to their moderate alkalinity. Incorporating Met into MCSNs may therefore provide a complementary strategy to overcome bacterial alkaline adaptation and further enhance antimicrobial efficacy.

Therefore, in this study, Met-loaded MCSNs were developed as a multifunctional intracanal therapeutic platform for the management of persistent endodontic infections. The physicochemical properties, Met-loading characteristics, release behavior, and biocompatibility of MCSNs-Met were systematically characterized. Furthermore, the antibacterial efficacy of MCSNs-Met against highly alkaline-resistant *E. faecalis* was evaluated using an infected dentin biofilm model and further validated in a clinically relevant beagle dog model of apical periodontitis. Collectively, this study presents a biomaterial-based therapeutic strategy that integrates enhanced microbial eradication with bioactive periapical tissue repair, offering a potential approach for addressing persistent root canal infections.

## Methods

### Synthesis of Met-loaded MCSNs

MCSNs were synthesized according to a previously reported method by Fan et al. [19]. Briefly, 660 mg of cetyltrimethylammonium bromide (CTAB; Sigma-Aldrich, USA) and 12 mL of ammonia solution were dissolved in 600 mL of deionized water under continuous magnetic stirring for 30 min. Subsequently, 30 mL of tetraethyl orthosilicate (TEOS; Aladdin Industrial Corporation, China) and 31.21 g of calcium nitrate tetrahydrate (Sinopharm Chemical Reagent Co., Ltd., China) were sequentially added to the solution. The mixture was vigorously stirred for 3 h to allow the formation of calcium silicate precursors. The resulting precipitates were collected by filtration, thoroughly washed three times with deionized water and absolute ethanol, and dried at 60 °C overnight. The dried powders were subsequently calcined in a muffle furnace at 550 °C for 6 h to remove the CTAB template, yielding MCSNs.

MCSNs-Met were prepared using a chemical immersion method as previously described [21]. Briefly, 1 g of MCSNs and 30 g of metformin hydrochloride (Aladdin, China) were dispersed in 1 L of deionized water under continuous stirring. The pH of the suspension was adjusted to approximately 7.0 by dropwise addition of 0.1 M HCl, followed by vigorous stirring at room temperature for 4 h. The suspension was then filtered, and the collected precipitates were washed three times with absolute ethanol to remove residual unbound Met. Finally, the obtained powders were dried at 60 °C overnight to obtain MCSNs-Met.

### Observation of the morphology of nanoparticles

A small amount of MCSNs and MCSNs-Met powders was individually dispersed onto conductive carbon tape and sputter-coated with a thin layer of gold at 30 mA for 120 s to enhance electrical conductivity. The surface morphology and particle size distribution of the nanoparticles were subsequently examined using field-emission scanning electron microscopy (FE-SEM; Zeiss, Germany).

### Observation of the internal structure of nanoparticles

A small amount of MCSNs and MCSNs-Met powders was individually dispersed in 1 mL of absolute ethanol and ultrasonicated for 10 min to obtain a homogeneous suspension. A drop of the suspension was then deposited onto a copper grid and air-dried at room temperature. The internal morphology and structural characteristics of the nanoparticles were subsequently examined using transmission electron microscopy (TEM; JEM-2100, Japan).

### Determination of specific surface area, pore volume, and pore size distribution

Prior to analysis, MCSNs and MCSNs-Met powders were vacuum-dried and degassed at 120 °C for 12 h to remove residual moisture and surface-adsorbed contaminants. Nitrogen adsorption-desorption isotherm measurements were conducted using an automated surface area and porosity analyzer (ASAP 2460, Micromeritics, USA). The specific surface area (S_BET_), pore volume (V_P_), and average pore diameter (D_P_) were calculated based on the Brunauer-Emmett-Teller (BET) and Barrett-Joyner-Halenda (BJH) models, respectively.

### Analysis of the zeta potential of nanoparticles

The surface charge characteristics of MCSNs and MCSNs-Met were determined by zeta potential analysis. Briefly, 10 mg of each nanoparticle sample was dispersed in 10 mL of deionized water and ultrasonicated for 10 min to obtain homogeneous suspensions. The suspensions were equilibrated at room temperature for 30 min before measurement. The zeta potential values were measured using a Zetasizer Nano ZS90 (Malvern Instruments, UK) at 25 °C.

### Analysis of the chemical structure of nanoparticles

The dried samples were thoroughly mixed with potassium bromide and compressed into pellets. Fourier transform infrared (FTIR) spectra were collected over the wavenumber range of 400–4000 cm^−1^ at a spectral resolution of 4 cm^−1^ using a Fourier transform infrared spectrometer (Nicolet 5700, Thermo Fisher Scientific, USA) to characterize the chemical composition and possible interactions of the nanoparticles.

### pH measurement of nanoparticles

A total of 400 mg of MCSNs or MCSNs-Met powder was immersed in 20 mL of deionized water and incubated at 37 °C under static conditions. The pH values of the suspensions were measured on days 1, 3, 6, and 9 using a calibrated pH meter (Sartorius AG, Germany).

### Release analysis of MCSNs-Met

A total of 400 mg of MCSNs-Met powder was immersed in 20 mL of deionized water and incubated at 37 °C. At predetermined time points (1, 3, 6, and 9 days), 1 mL of the supernatant was collected and filtered through a 0.22-μm membrane filter (Merck Millipore, Germany). Subsequently, an equal volume of fresh deionized water was added to maintain a constant volume. The absorbance of the collected samples was measured at 232 nm using a UV-Vis spectrophotometer (UV-2401PC, Kyoto, Japan), and the concentration of released Met was calculated based on an external standard calibration curve.

### Cytotoxicity evaluation

Briefly, 100 mg of MCSNs-Met nanoparticles were immersed in 1 mL of *α*-minimum essential medium (Thermo Fisher Scientific, USA) supplemented with 1% penicillin/streptomycin (Thermo Fisher Scientific, USA) and incubated at 37 °C for 24 h. The resulting extracts were sterilized by filtration through a 0.22-μm membrane filter (Merck Millipore, Darmstadt, Germany) and supplemented with 10% fetal bovine serum (Thermo Fisher Scientific, USA). Extracts with different concentrations (20, 50, and 100 mg/mL) were prepared for subsequent experiments. MC3T3-E1 cells, a mouse calvaria-derived preosteoblastic cell line (American Type Culture Collection, ATCC, USA), were seeded into 96-well plates at a density of 1 × 10^5^ cells/well and cultured for 24 h. This cell line was selected to evaluate the cytocompatibility of MCSNs-Met as osteoblast-lineage cells are relevant to the periapical bone repair. Subsequently, the original culture medium was replaced with the prepared extraction media. Cell viability was assessed after 3, 6, and 9 days using the Cell Counting Kit-8 (CCK-8; Kumamoto, Japan) assay according to the manufacturer’s instructions. The absorbance was measured at 450 nm using a microplate reader (Synergy H1, BioTek, USA).

### Dentinal tubule penetration of MCSNs-Met and CHM

The protocol for the dentin experiment in this study has been approved by the Ethics Committee of the School and Hospital of Stomatology, Wuhan University (No. [2022] A20). MCSNs-Met nanoparticles and calcium hydroxide/metformin paste (CHM) were labeled with Rhodamine B (RhB) using the same protocol to ensure comparable fluorescence intensity and eliminate potential interference caused by different RhB concentrations. Briefly, MCSNs-Met nanoparticles were dispersed in 0.05% (w/w) RhB (Aladdin, Shanghai, China) aqueous solution and stirred under dark conditions for 12 h. The suspension was centrifuged and repeatedly washed with deionized water until no obvious fluorescence was detected in the supernatant to remove unbound RhB. The obtained RhB-labeled MCSNs-Met nanoparticles were resuspended in sterile saline at a concentration of 20 mg/mL before use. Similarly, CHM was labeled with RhB by dispersing the CHM powder in a 0.05% (w/w) RhB aqueous solution under identical dark stirring conditions for 12 h. After repeated washing to remove unbound RhB, the RhB-labeled CHM powder was mixed with sterile saline to prepare a paste with an equivalent RhB concentration to that of the RhB-labeled MCSNs-Met suspension.

Extracted human single-rooted mandibular premolars without caries, cracks, root resorption, calcification, or additional canals were selected. Root canals were prepared using the Reciproc 25 system (VDW GmbH, Germany), and the roots were standardized to 12 mm in length after crown removal. The canals were irrigated with 2% NaOCl and 17% EDTA during and after instrumentation to remove the smear layer, followed by apical sealing with flowable resin (SHOFU, Japan). The RhB-labeled CHM paste and RhB-labeled MCSNs-Met suspension were introduced into the prepared root canals and ultrasonically activated for 1 min using a P5 Newtron ultrasonic device (Acteon, France) at power setting 4. The roots were then sectioned into three equal parts using a low-speed precision cutter (Buehler, USA). The buccal and lingual surfaces of each section were examined by confocal laser scanning microscopy (CLSM; Leica, Germany). RhB fluorescence was detected at excitation/emission wavelengths of 555/588 nm. ImageJ software (Bethesda, USA) was used to quantify the fluorescence penetration depth.

### Antibiofilm activity of MCSNs-Met against highly alkaline-resistant E. faecalis biofilms on dentin slices

A dentin biofilm model was constructed using highly alkaline‑resistant *E. faecalis* XS 003, which was isolated from the root canal of teeth with failed root canal treatment, as described in our previous study [12]. Briefly, biofilm-infected dentin slices were treated with MCSNs or MCSNs-Met suspensions at equivalent concentrations. Untreated infected dentin slices incubated with sterile saline were used as the biofilm control group. After 24 h of treatment, the antibacterial efficacy was evaluated by FE-SEM (Zeiss, Germany) and CLSM using the LIVE/DEAD BacLight Bacterial Viability Kit (Thermo Fisher Scientific, USA). To further assess the regrowth capacity of residual biofilm-associated bacteria, bacteria were recovered from the treated dentin slices and cultured in fresh medium. Bacterial regrowth was monitored by measuring the optical density at 600 nm (OD600) at 3, 6, 9, 12, and 24 h, as previously described [12].

### Establishment and treatment of a beagle model of apical periodontitis induced by highly alkaline-resistant E. faecalis

A total of six healthy male beagles (12–18 months old, 9–11 kg) were obtained from the Hubei Provincial Center for Disease Control and Prevention. All animal procedures were approved by the Animal Ethics Committee of Hubei Provincial Center for Disease Control and Prevention (No. 202450112). Each beagle contributed eight premolars (bilateral maxillary and mandibular second and third premolars), yielding a total of 48 teeth. Among these, eight teeth were randomly assigned as healthy controls and received no endodontic intervention. The remaining 40 teeth were subjected to experimental induction of apical periodontitis using highly alkaline-resistant *E. faecalis* XS 003 and subsequently randomly allocated into five treatment groups (*n* = 8 teeth/group). The experimental groups consisted of five apical periodontitis treatment groups: NS (normal saline), MCSNs (20 mg/mL MCSNs suspension), CH (calcium hydroxide paste, powder at a ratio of 1:2 with sterile saline), CHM (calcium hydroxide/metformin mixture [12], CHM powders at a ratio of 1:2 with sterile saline), and MCSNs-Met (20 mg/mL MCSNs-Met suspension).

After general anesthesia was induced by intramuscular injection of a mixture of Sumianxin II and Zoletil 50, anesthesia was maintained with 1.5–3% isoflurane inhalation in oxygen. Vital signs were continuously monitored throughout the procedure. Access cavities were prepared under an operating microscope, and the pulp tissue was removed. A sterile cotton pellet saturated with an *E. faecalis* XS 003 suspension (1 × 10^8^ CFU/mL) was then placed into the pulp chamber. The access cavities were subsequently sealed with a flowable resin (SHOFU, Japan). After 3 weeks, periapical radiographs were obtained, and the presence of periapical radiolucency was considered indicative of successful establishment of the apical periodontitis model.

Following successful establishment of the apical periodontitis model, the root canals were negotiated using a #10 K-file, and the working length was determined using an electronic apex locator (PROPEX II, Dentsply Sirona, USA). Root canal preparation was performed to size F3 using ProTaper nickel-titanium rotary instruments (Dentsply Sirona, USA). During instrumentation, the canals were irrigated alternately with 5.25% sodium hypochlorite and sterile saline. After placement of the intracanal medicaments, ultrasonic activation was performed using a Satelec P5 Newtron ultrasonic device (Acteon, France) at a power setting of 4 for 1 min. The volume of intracanal medicaments was standardized across groups. The access cavities were subsequently sealed with an adhesive system and flowable resin.

Three weeks after intracanal medication, the animals were euthanized under deep anesthesia. The specimens were perfused through the common carotid artery with 4 L of saline, followed by 4 L of 4% paraformaldehyde (Servicebio, China). The jaw specimens were harvested and further fixed in 4% paraformaldehyde at 4 °C for 7 days prior to subsequent analyses.

### Micro-computed tomography (Micro-CT) analysis

The specimens were scanned using a Micro-CT scanner (SCANCO MEDICAL μCT50, Switzerland). The scanning parameters were set as follows: a tube voltage of 70 kV, a current of 114 μA, and an isotropic voxel size of 30 μm. The acquired datasets were reconstructed into three-dimensional (3D) images, and the volume of periapical bone loss was quantitatively calculated using ImageJ software (Bethesda, USA) and Mimics Research 19.0 software (Materialise NV, Belgium).

### Preparation of periapical histological sections from beagle specimens

Following Micro-CT analysis, the specimens were decalcified in 10% ethylenediaminetetraacetic acid solution at room temperature for 5 months under continuous agitation, with the decalcifying solution replaced every other day. After complete decalcification, the specimens were rinsed overnight with running water, dehydrated through a graded ethanol series, embedded in paraffin, and longitudinally sectioned at a thickness of 5 μm.

### Gram, hematoxylin–eosin (HE), and Masson’s trichrome staining

Paraffin-embedded sections were subjected to Gram staining according to the manufacturer’s instructions (Servicebio, China) to evaluate bacterial distribution within the root canal system. HE staining was performed to assess inflammatory responses and morphological changes in the periapical tissues. Masson’s trichrome staining was conducted to evaluate collagen deposition and the extent of periapical tissue repair. All stained sections were digitized using an Aperio ScanScope CS scanner (Aperio, USA).

### Tartrate-resistant acid phosphatase (TRAP) staining

Paraffin sections were deparaffinized, rehydrated, and subjected to TRAP staining using a staining kit (Servicebio, China) according to the manufacturer’s instructions. The sections were subsequently counterstained with hematoxylin. TRAP-positive multinucleated osteoclasts located along the bone surface or within resorption lacunae were identified and quantified using CaseViewer software (3DHISTECH, Hungary).

### Statistical analysis

Statistical analyses were performed using GraphPad Prism 8 and SPSS software. For animal experiments, teeth were considered nested within individual animals. Accordingly, linear mixed-effects models were applied for comparisons among infected treatment groups, with treatment group as a fixed effect and animal identity as a random effect to account for intra-animal correlations. Healthy controls were excluded from therapeutic efficacy comparisons and were used only as physiological references. Estimated marginal means were compared using Tukey-adjusted pairwise comparisons. All *in vitro* experiments were performed with at least three independent biological replicates. For *in vitro* experiments, one-way or two-way analysis of variance (ANOVA) followed by Tukey’s multiple comparison test were performed according to the experimental design. When assumptions of normality or homogeneity of variance were not satisfied, Kruskal-Wallis tests followed by Dunn’s multiple comparison tests were applied. Data distribution and model assumptions were examined before statistical analyses. Data are presented as mean ± standard deviation (SD), and *p* < 0.05 was considered statistically significant.

## Results

### Physicochemical characteristics of MCSNs-Met

FE-SEM and TEM images revealed that both MCSNs and MCSNs-Met exhibited a relatively uniform spherical morphology, with particle sizes of approximately 110 nm, as visually estimated from representative TEM images (Figure 1A). TEM analysis further confirmed the presence of well-defined mesoporous structures in both nanoparticles (Figure 1A). Nitrogen adsorption-desorption analysis showed typical type IV isotherms for both MCSNs and MCSNs-Met, indicating their mesoporous characteristics (Figure 1B). Pore size distribution analysis revealed characteristic mesoporous structures in both MCSNs and MCSNs-Met, with the main distribution peaks located at approximately 3 nm (Figure 1C). Furthermore, MCSNs-Met exhibited decreased specific surface area, pore volume, and average pore diameter compared with MCSNs (Table 1), further supporting successful occupation of the mesoporous structure by Met molecules. Zeta potential analysis revealed that MCSNs exhibited a negative surface charge, whereas MCSNs-Met showed a significantly reduced negative charge after Met incorporation, indicating successful interaction between Met molecules and the MCSNs surface (Figure 1D).

**Figure 1.**
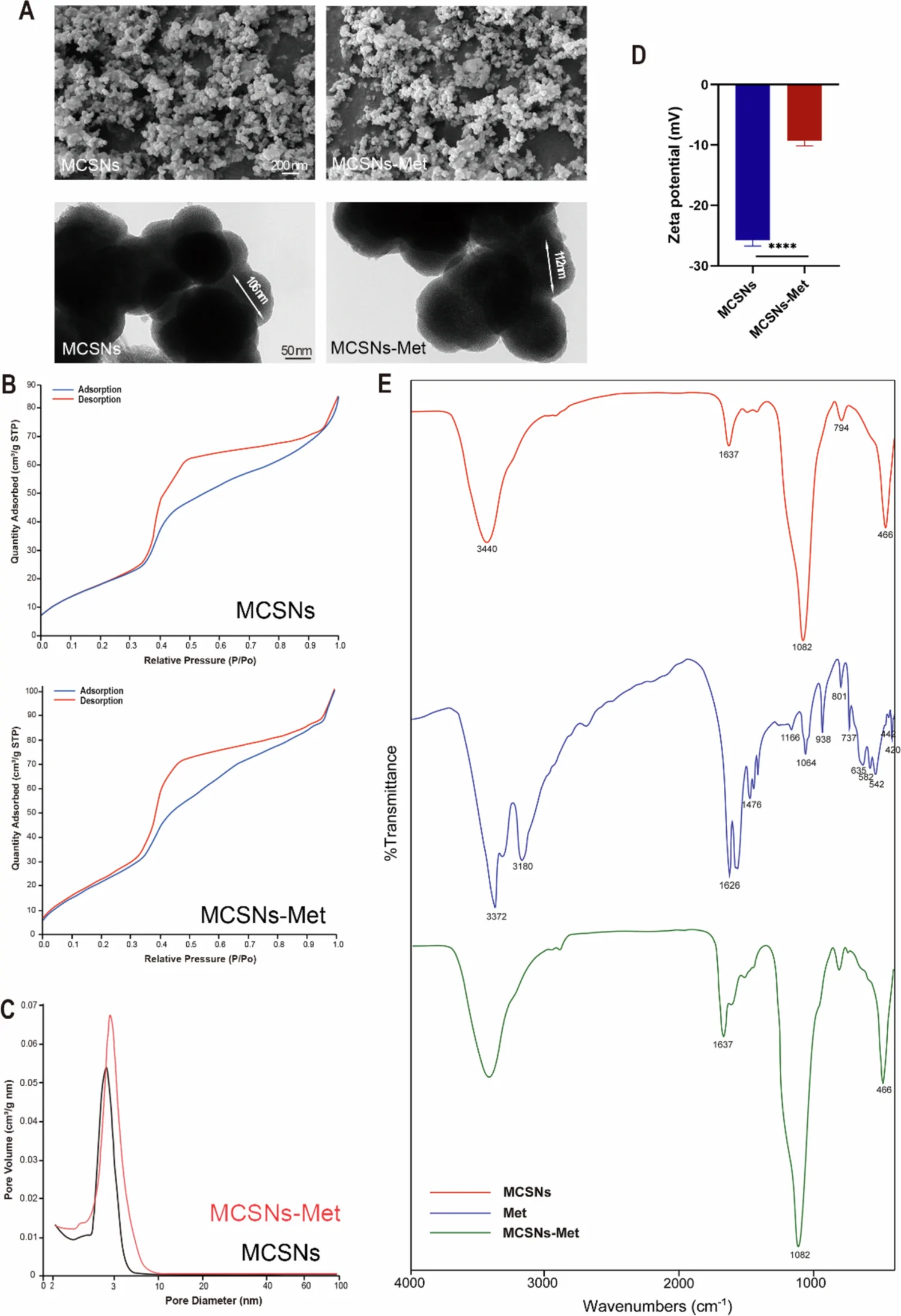
Physicochemical characteristics of MCSNs-Met. (A) Representative FE-SEM and TEM images of MCSNs and MCSNs-Met. (B) Nitrogen adsorption-desorption isotherms of MCSNs and MCSNs-Met. (C) Pore size distribution of MCSNs and MCSNs-Met. (D) Zeta potential of MCSNs and MCSNs-Met. (E) FTIR spectra of MCSNs, Met and MCSNs-Met. Statistical analysis of (D) was performed using one-way ANOVA followed by Tukey's multiple comparisons test. ****p < 0.0001.

**Table 1.** Representative textural parameters of MCSNs and MCSNs-Met.

Nanoparticle	S_BET_ (m^2^g^−1^)	V_P_ (cm^3^g^−1^)	D_P_ (nm)
MCSNs	122.34	0.19	7.16
MCSNs-Met	55.05	0.11	4.52

FTIR spectra further confirmed the successful loading of Met into MCSNs (Figure 1E). The broad band at approximately 3370–3400 cm^−1^ was attributed to O-H stretching vibrations from surface hydroxyl groups and adsorbed water molecules. The characteristic bands at 1082, 794, and 466 cm^−1^ corresponded to asymmetric stretching, symmetric stretching, and bending vibrations of Si-O-Si bonds, respectively, confirming the presence of the calcium silicate framework. Met exhibited characteristic peaks associated with its biguanide structure, including N-H stretching vibrations (3372–3180 cm^−1^), N-H bending vibrations (1637 cm^−1^), and C-N stretching vibrations (1166–1060 cm^−1^). Compared with MCSNs, MCSNs-Met displayed additional Met-associated peaks and altered peak intensities, suggesting successful Met incorporation and possible intermolecular interactions between Met and the MCSNs framework.

### Dentinal tubule penetration, release behavior, biocompatibility, and antibacterial activity against E. faecalis biofilms of MCSNs-Met

Quantitative analysis of dentinal tubule penetration revealed that RhB-labeled MCSNs-Met exhibited deeper infiltration into dentinal tubules compared with CHM across all root regions (Figure 2A, B). Dynamic pH monitoring revealed that both MCSNs and MCSNs-Met maintained an alkaline environment over 9 days. The pH values of MCSNs and MCSNs-Met remained approximately 10.0 and 10.5, respectively, with the slightly higher alkalinity of MCSNs-Met potentially attributed to the intrinsic alkaline properties of Met (Figure 2C). The cumulative release profile demonstrated a sustained increase in Met release from MCSNs-Met over 9 days, indicating its prolonged drug-delivery capability (Figure 2D). CCK-8 assays showed that MCSNs-Met at concentrations up to 100 mg/mL did not significantly reduce MC3T3-E1 cell viability, indicating good cytocompatibility (Figure 2E).

**Figure 2.**
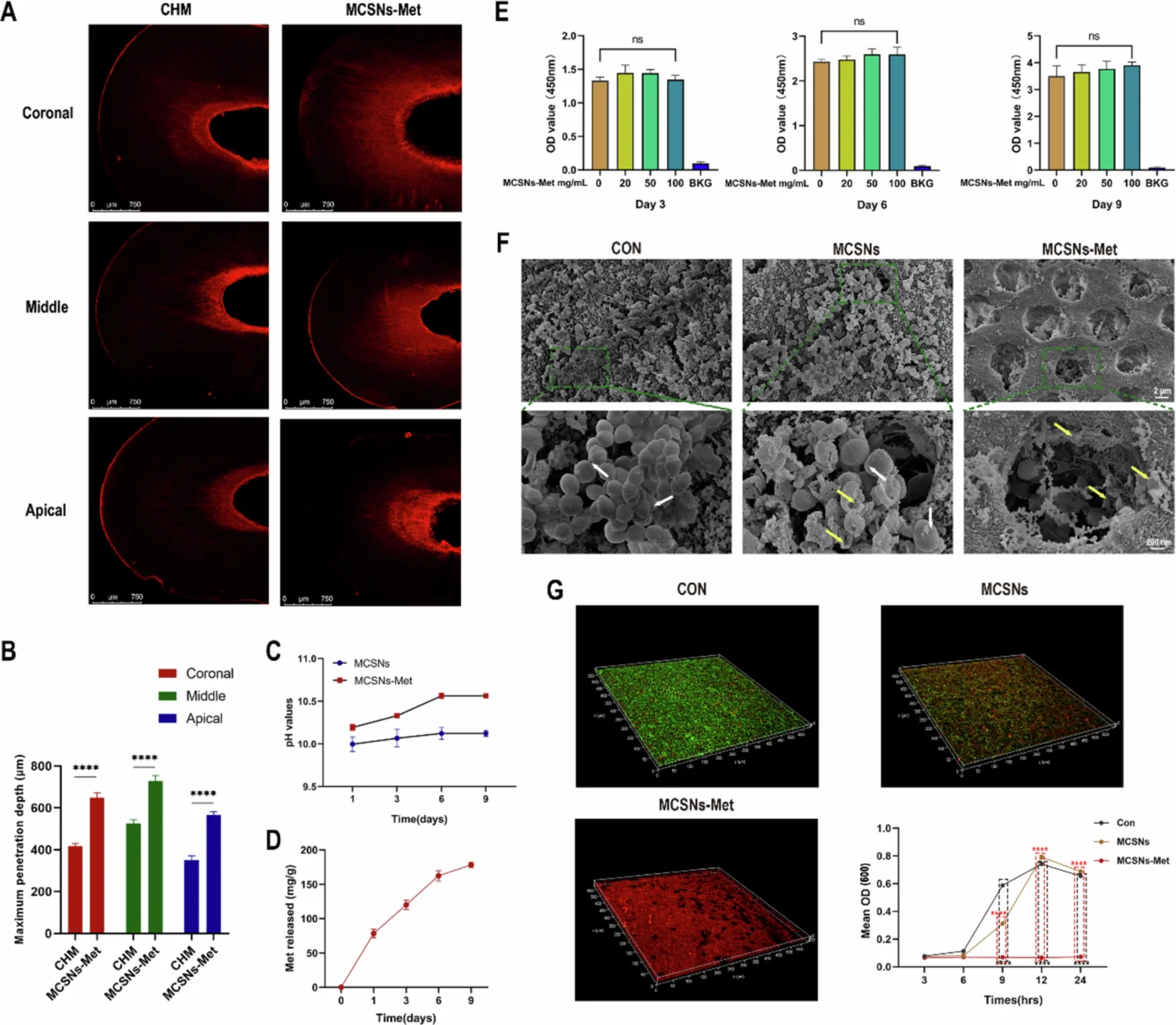
Penetration ability, Antibiofilm activity, pH profile, Met release profile, and biocompatibility of MCSNs-Met. (A) Representative CLSM images showing dentinal tubule penetration of RhB‑labeled CHM and MCSNs‑Met within the coronal, middle, and apical root‑canal regions. (B) Dentinal tubule penetration depth of CHM and MCSNs-Met. (C) pH dynamic monitoring of MCSNs and MCSNs-Met. (D) Cumulative Met release from MCSNs-Met. (E) Cytocompatibility of MCSNs-Met evaluated by CCK-8 assays, BKG (background blank). (F) Representative FE-SEM images showing *E. faecalis* biofilm morphology following different treatments. CON, infected dentin treated with sterile saline. Green dashed boxes indicate regions shown at higher magnification in the corresponding lower panels. White arrows indicate intact bacterial cells, while the yellow arrow highlights damage to the biofilm structure and disruption of bacterial structures after treatment. (G) CLSM images showing live (green) and dead (red) bacteria, together with dynamic growth monitoring of residual bacteria after treatment. CON, infected dentin treated with sterile saline. Statistical analyses were performed using two-way repeated-measures ANOVA for (B, G), one-way ANOVA was performed separately at each time point for (E). Data are presented as mean ± SD. ns, not significant (*p* ≥ 0.05); ****p < 0.0001.

FE-SEM images showed that dense biofilms remained on the dentin surfaces treated with MCSNs, with most bacterial cells maintaining intact morphology and bacterial aggregates observed within dentinal tubules. In contrast, MCSNs-Met treatment markedly disrupted the biofilm architecture, resulting in fragmented bacterial structures and reduced bacterial adhesion on the dentin surface and within dentinal tubules (Figure 2F). CLSM analysis revealed fewer live bacterial fluorescence signals following MCSNs-Met treatment than following MCSNs treatment. In addition, OD600-based growth monitoring revealed significantly reduced regrowth of residual bacteria recovered from MCSNs-Met-treated dentin biofilms compared with the control and MCSNs groups at 9, 12, and 24 h (Figure 2G). These findings suggest that MCSNs-Met impaired the regrowth capacity of residual biofilm-associated bacteria.

### Therapeutic effects of MCSNs-Met on apical periodontitis in beagles

The therapeutic efficacy of MCSNs-Met was evaluated in a beagle model of apical periodontitis induced by highly alkaline-resistant *E. faecalis* XS 003. Micro-CT analysis demonstrated extensive periapical bone destruction in the NS, MCSNs, and CH groups. Compared with these groups, CHM treatment reduced periapical bone loss, whereas the MCSNs-Met group exhibited the most pronounced reduction in periapical bone resorption volume (Figure 3A).

**Figure 3.**
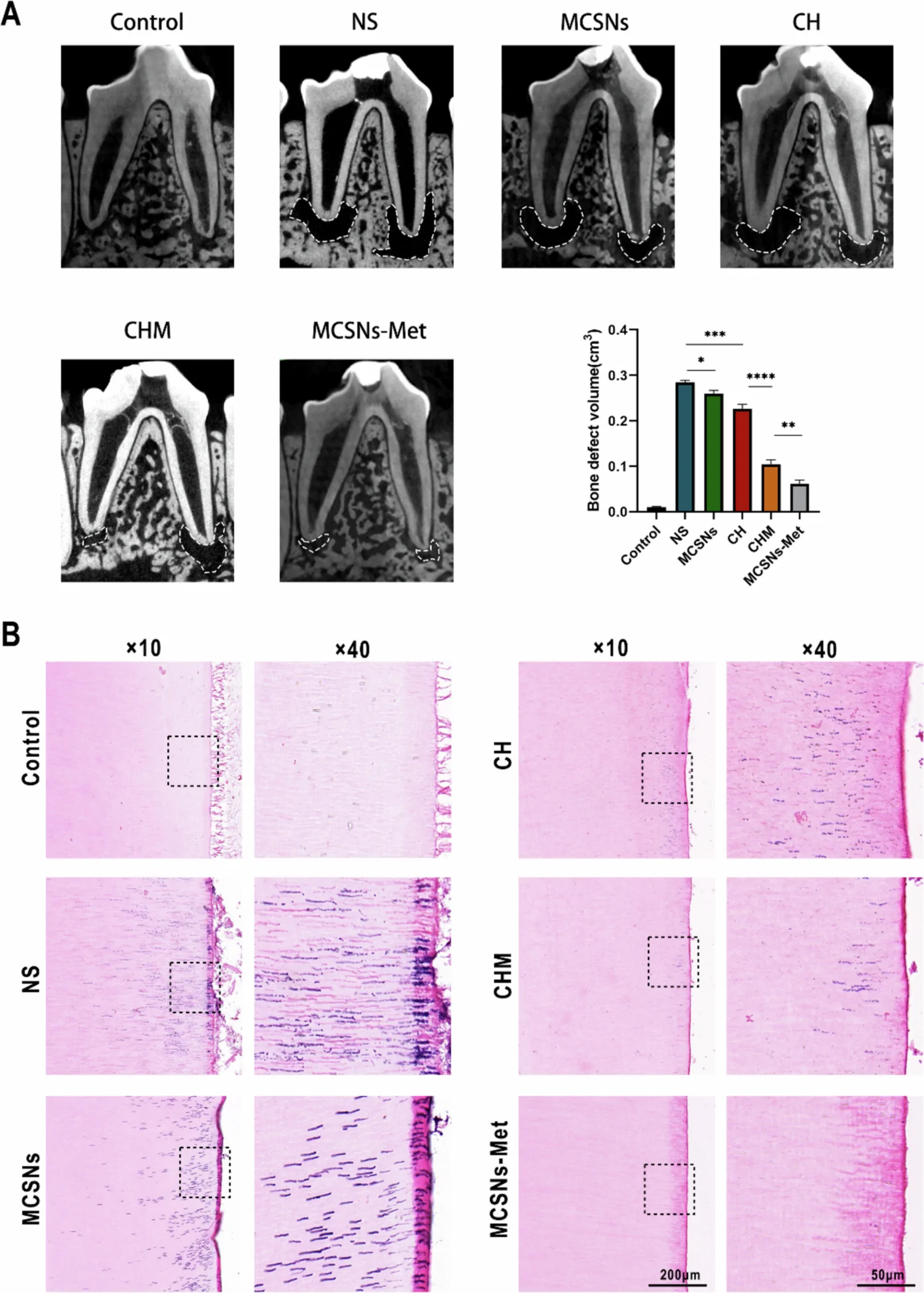
Effect of intracanal treatments on periapical bone resorption and residual bacterial colonization in *E. faecalis*-induced apical periodontitis in beagle dogs. (A) Representative Micro-CT images and quantitative analysis of periapical bone resorption. Areas marked with a white dashed line indicate periapical bone defects. (B) Representative Gram-stained sections showing residual bacterial colonization within dentinal tubules at ×10 and ×40 magnification. Dashed boxes in the ×10 images indicate the regions shown at higher magnification (×40). Control, healthy uninfected teeth without endodontic intervention; NS, infected teeth treated with normal saline; MCSNs, infected teeth treated with MCSNs; CH, infected teeth treated with calcium hydroxide; CHM, infected teeth treated with CHM; MCSNs-Met, infected teeth treated with MCSNs-Met. Statistical analysis of (A) was performed using a linear mixed-effects model with treatment as a fixed effect and animal identity as a random effect, followed by Tukey-adjusted pairwise comparisons among infected groups. *p < 0.05; **p < 0.01; ***p < 0.001; ****p < 0.0001.

Gram staining revealed abundant Gram-positive bacterial colonization within dentinal tubules in the NS, MCSNs, and CH groups. Although bacterial infiltration was reduced after CHM treatment, residual Gram-positive bacteria remained detectable in the middle and apical thirds of root canals. In contrast, minimal bacterial signals were observed in the MCSNs-Met group (Figure 3B).

HE staining showed severe inflammatory infiltration and periapical tissue destruction in the NS, MCSNs, and CH groups. The CHM group exhibited reduced inflammatory responses, while MCSNs-Met treatment further alleviated inflammatory infiltration and improved periapical tissue morphology (Figure 4A). Masson’s trichrome staining demonstrated enhanced collagen deposition and extracellular matrix remodeling in the MCSNs-Met group, indicating improved periapical tissue repair (Figure 4A). Furthermore, TRAP staining revealed significantly fewer TRAP-positive osteoclasts in the MCSNs-Met group compared with the CHM group, whereas increased osteoclast activation was observed in the NS and conventional treatment groups (Figure 4B).

**Figure 4.**
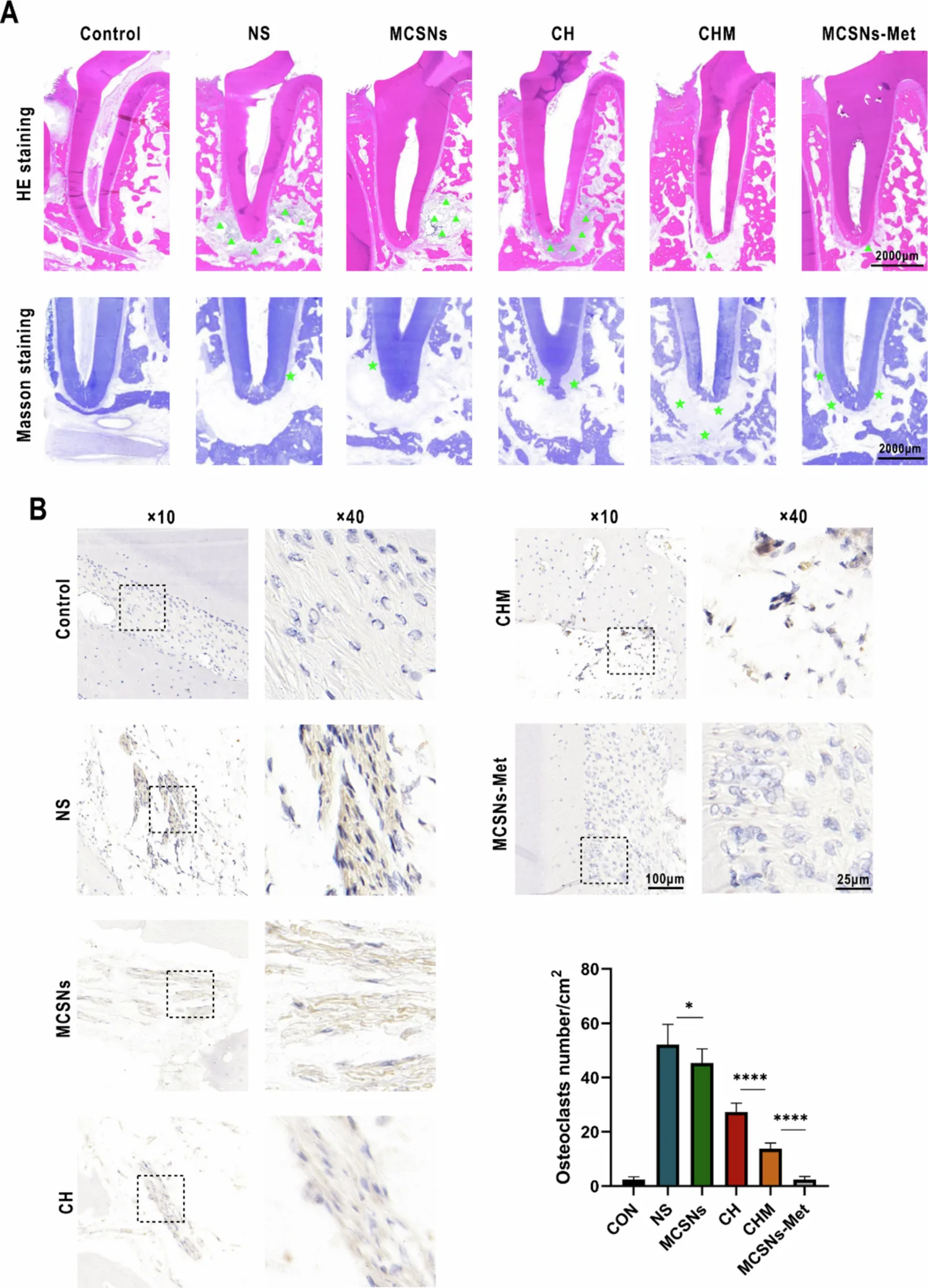
Effects of intracanal treatments on periapical inflammation, tissue repair, and osteoclast activation in apical periodontitis in beagle dogs. (A) Representative HE and Masson’s trichrome staining images showing periapical inflammatory responses, tissue morphology, and collagen deposition following different intracanal treatments. Green triangles indicate areas of inflammatory cell infiltration in HE-stained sections, while green pentagrams highlight collagen-rich regions in Masson's trichrome-stained sections. (B) Representative TRAP-stained sections and quantitative analysis of TRAP-positive osteoclasts in periapical tissues. Dashed boxes in the ×10 images indicate the regions shown at higher magnification (×40). Control, healthy uninfected teeth without endodontic intervention; NS, infected teeth treated with normal saline; MCSNs, infected teeth treated with MCSNs; CH, infected teeth treated with calcium hydroxide; CHM, infected teeth treated with CHM; MCSNs-Met, infected teeth treated with MCSNs-Met. Statistical analysis of the osteoclast counts in (B) was performed using a linear mixed-effects model with treatment as a fixed effect and animal identity as a random effect, followed by Tukey-adjusted pairwise comparisons among infected groups. *p < 0.05; ****p < 0.0001.

## Discussion

Persistent microorganisms residing within dentinal tubules remain a major challenge in endodontic therapy, as these anatomical niches provide protection from conventional antimicrobial strategies and contribute to persistent infection and treatment failure [3]. Although conventional intracanal medicaments, particularly CH, exhibit potent antibacterial activity, highly alkaline-resistant microorganisms such as *E. faecalis* can survive under these harsh conditions and contribute to persistent apical periodontitis [3,7]. In our previous study, we demonstrated that Met enhanced the susceptibility of alkaline-resistant *E. faecalis* to CH by disrupting bacterial metabolic adaptation mediated by the mannose phosphotransferase system (Man-PTS EII), thereby improving the biofilm-eradicating efficacy of CH against *E. faecalis* [12]. Although CHM can effectively eliminate superficial dentin biofilms and promote periapical healing in rat models, its limited penetration into dentinal tubules and insufficient long-term therapeutic activity restrict its ability to eradicate deep-seated microorganisms and facilitate effective periapical tissue repair. Thus, effective microbial control requires not only potent antibacterial activity but also sustained therapeutic effects within the complex dentin microenvironment. Developing an effective delivery platform capable of enhancing Met retention, facilitating intradentinal penetration, and enabling sustained alkaline antibacterial activity therefore represents a critical step toward overcoming the translational limitations of Met-based intracanal therapy.

Accordingly, MCSNs were selected not only as a Met delivery carrier but also as an intrinsically bioactive component for intracanal therapy. Their favorable physicochemical properties, including a high specific surface area, well-defined mesoporous architecture, and excellent drug-loading and controlled-release capabilities, provide an effective platform for Met incorporation and sustained local delivery [22]. The interaction between MCSNs and Met is primarily governed by the protonation state of Met and the surface charge characteristics of the nanoparticles [21,23]. Under neutral conditions, Met predominantly exists in a monoprotonated form, which may facilitate electrostatic interactions with negatively charged sites on the MCSN surface, thereby promoting Met adsorption within the mesoporous structure [21,24]. Moreover, the stable mesoporous framework of MCSNs provides a suitable reservoir for Met incorporation, restricting rapid diffusion of the drug and enabling sustained release within the root canal environment. Importantly, MCSNs themselves possess intrinsic alkaline antibacterial activity, which may contribute to bacterial suppression within the root canal microenvironment. In addition, ultrasonic activation can enhance the dispersion and penetration of MCSNs into complex root canal anatomies and dentinal tubules, thereby facilitating their localized antimicrobial effects and improving Met delivery to bacteria residing in these anatomical niches. Compared with CHM treatment, MCSNs-Met therefore integrates the intrinsic alkaline antibacterial activity and dentinal tubule accessibility of MCSNs with the sustained delivery and antimicrobial activity of Met, potentially prolonging bacterial exposure to an unfavorable alkaline environment and enhancing the susceptibility of highly alkaline-resistant *E. faecalis* to antimicrobial treatment.

Successful management of apical periodontitis involves more than microbial elimination; effective modulation of inflammation and restoration of damaged periapical tissues are also essential for favorable long-term outcomes [25,26]. In addition to their role in Met delivery, MCSNs possess intrinsic bioactivity derived from calcium and silicate ion release, which can promote mineralized matrix formation and osteogenic responses [19,20]. Consistent with this rationale, MCSNs-Met demonstrated enhanced bacterial elimination, promoted mineralized tissue formation, facilitated periapical tissue repair, and reduced osteoclast activity in the periapical region. These findings indicate that MCSNs function as more than a passive drug carrier; instead, they act as a bioactive therapeutic component that contributes to both antimicrobial enhancement and tissue regeneration. Together, these findings support the potential of MCSNs-mediated Met delivery as an integrated antimicrobial and regenerative strategy.

To further confirm the translational potential of MCSNs-Met, its therapeutic efficacy was evaluated in a beagle model of apical periodontitis, which provides a clinically relevant platform for assessing intracanal therapeutic strategies [27]. Although rodent models are widely used for investigating antibacterial mechanisms and preliminary regenerative effects, their small tooth size, narrow root canals, and limited anatomical complexity present challenges for standardized root canal preparation and intracanal medicament delivery, potentially restricting the clinical extrapolation of findings [28,29]. In contrast, beagle teeth exhibit greater anatomical and procedural similarities to human teeth, including more comparable root canal dimensions, complex root morphology, and feasibility for clinically relevant endodontic procedures [30]. Therefore, the beagle model used in this study provided an important translational step toward evaluating the clinical potential of MCSNs-Met, further supporting the therapeutic promise of MCSNs-Met for the management of apical periodontitis.

Several limitations of this study should be acknowledged. First, although MCSNs-Met exhibited sustained Met release *in vitro*, dynamic assessment of drug release kinetics and local retention behavior within the root canal environment will be necessary to further elucidate its pharmacological performance. Second, because apical periodontitis is a polymicrobial disease involving complex microbial communities, future studies incorporating multispecies biofilm models and clinically representative infection systems are needed to further validate the antibacterial efficacy of MCSNs-Met. Furthermore, although the beagle model was established using a defined *E. faecalis* strain, the potential presence of other oral microorganisms within the root canal system cannot be excluded. Gram staining cannot distinguish *E. faecalis* from other bacterial species; therefore, future studies using species-specific molecular detection or microbial sequencing are needed to further characterize the residual bacterial communities. Finally, the compatibility of MCSNs-Met with subsequent root canal obturation procedures, as well as its long-term biosafety and therapeutic stability, requires further evaluation before clinical translation.

Collectively, this study establishes a mechanism-guided MCSNs-mediated Met delivery strategy that integrates sustained antimicrobial activity with the regenerative potential of calcium silicate, overcoming the limitations of conventional Met administration within the root canal microenvironment. MCSNs-Met effectively inhibited alkaline-resistant *E. faecalis* biofilms and promoted periapical tissue healing in a clinically relevant beagle model of apical periodontitis, highlighting its potential as a promising intracanal therapeutic strategy for persistent apical periodontitis (Figure 5).

**Figure 5.**
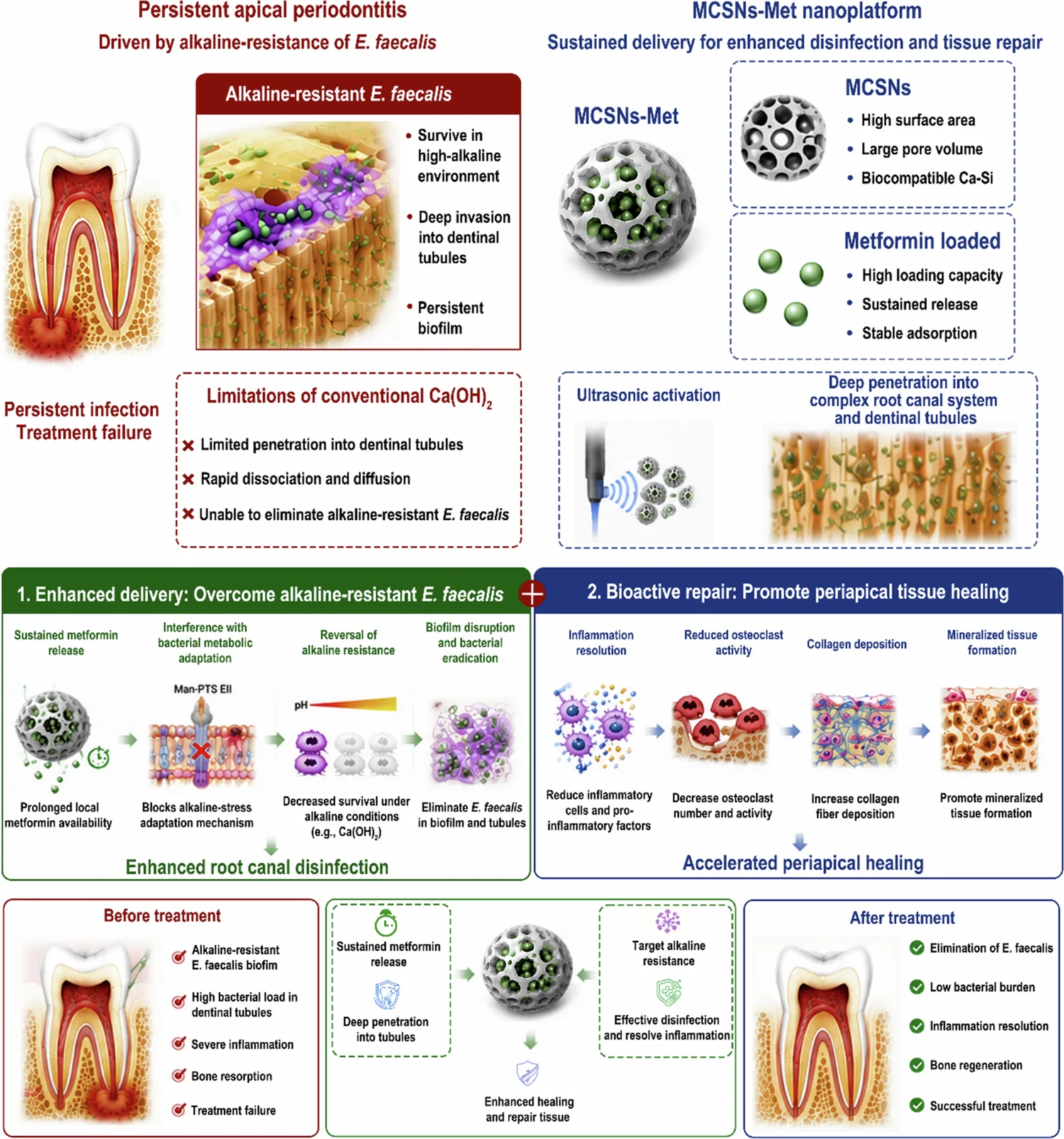
Schematic illustration of MCSNs-Met-mediated root canal disinfection and periapical healing in persistent apical periodontitis.

## Conclusion

MCSNs-Met provides a mechanism-guided intracanal therapeutic strategy that integrates the intrinsic alkalinity of MCSNs with the ability of Met to attenuate the alkaline resistance of *E. faecalis*. By maintaining a sustained alkaline microenvironment while simultaneously sensitizing highly alkaline-resistant *E. faecalis* to alkaline stress, MCSNs-Met achieved enhanced biofilm control and promoted periapical tissue healing in a clinically relevant beagle model of apical periodontitis. These findings support MCSNs-Met as a promising biomaterial-based intracanal medicament for the management of persistent apical periodontitis.

## Data Availability

The data that support the findings of this study are included in this published manuscript.
